# Right atrial isomerism in children older than 3 years

**DOI:** 10.1186/s40064-016-3007-6

**Published:** 2016-08-20

**Authors:** Sun Yan, Wang Jianpeng, Quan Xin, Zhang Minghui, Zhang Li, Wang Hao

**Affiliations:** Department of Echocardiography, Fuwai Hospital, Chinese Academy of Medical Sciences, Peking Union Medical College, No. 167, Bei Li Shi Road, Xicheng District, Beijing, 100037 China

## Abstract

**Background:**

There is a high mortality in infants with right atrial isomerism (RAI). However, less is known about outcome in older children with RAI. This study sought to evaluate those patients with RAI who survived older than 3 years of age without surgical intervention.

**Results:**

A total of 33 consecutive patients (20 males) were enrolled in the study, mean age 6 years (range 3–32). None of the patients had surgical intervention for the RAI before age 3. Cardiac abnormalities include altered cardiac position (39 %), atrioventricular valve anomaly (87 %), single or functional single ventricle (55 %), pulmonary/subpulmonary obstruction (97 %), abnormal origin of the aorta (100 %), bilateral superior vena cava (67 %), and anomalous pulmonary venous drainage (66 %). Surgical intervention was performed after 3 years of age in 20 patients (61 %). None of them planned or had biventricular repair performed. 10 patients underwent the total cavopulmonary connection procedure, including four (40 %) who had atrioventricular valve (AVV) repair at the same time [all with common atrioventricular valve (CAVV)]. One patient died the day after the operation. A total of 69 % of patients with a CAVV had moderate or severe regurgitation, while 27 % with a single atrioventricular valve had moderate or severe regurgitation.

**Conclusion:**

Patients with RAI who have survived to early childhood without surgical intervention have complex cardiac abnormalities. Survival after single stage total cavopulmonary connection is good but AVV repair is common.

## Background

Congenital heart disease (CHD) affects 0.75–0.9 % of newborns and is a leading cause of infant mortality (Van der Linde et al. 2011; Hoffman and Kaplan 2002). With major improvements in medical and surgical treatment of CHD in recent years, more than 90 % of patients with CHD survive into adulthood (Khairy et al. 2010; Marelli et al. 2007). However, heterotaxy syndrome is one of the most serious forms of CHD, occurring in approximately 1 in 5000–7000 live births with CHD (Reller et al. 2008; Lin et al. 2000).

The International Nomenclature Committee for Pediatric and Congenital Heart Disease defines heterotaxy syndrome as an abnormality where the internal thoraco-abdominal organs demonstrate abnormal arrangement across the left–right axis of the body (Jacobs et al. 2007). Patients with heterotaxy syndrome are subdivided into right atrial isomerism (RAI) and left atrial isomerism (LAI) (Cohen et al. 2007). RAI is typically associated with complex cardiovascular malformations (Freedom et al. 2005; Eronen et al. 2013), with overall 5-year survival rates ranging from 30 to 74 % (Sinzobahamvya et al. 1999; Culbertson et al. 1992). RAI has a particularly high mortality rate in infants. To our knowledge, there are no previous reports on older RAI patients who have not had surgery with regard to survival and how their cardiac condition differs from infants with RAI.

Diagnosis of RAI remains a challenge. Patients with RAI have abnormalities in the lungs and various intra-abdominal organs, and each side of the heart morphologically resembles the right atrium and right atrial appendages. Most affected patients also have juxtaposition of the abdominal aorta and asplenia. Radiological diagnosis of RAI may be suspected on plain radiographic films of the chest and abdomen, by identifying the position of the liver and stomach, and bronchial pattern (Freedom and Fellow 1973). Echocardiographic or angiographic demonstration of a juxtaposition of the aorta and inferior vena cava, and morphology of the atrial appendages are used to support the diagnosis (Huhta et al. 1982).

This study aimed to investigate the natural history of patients with RAI whose surgical correction did not occur until after 3 years old. We also sought to assess their surgical outcomes when late palliation was performed.

## Methods

### Definitions and identification of patients

We performed a retrospective study of all patients who were diagnosed between October 2010 and February 2014 with RAI in our pediatric center and were older than 3 years without prior surgical correction. A total of 33 consecutive patients (20 males, 13 females) were identified with a median age of 6 years (range: 3–32 years). Diagnosis of RAI was confirmed at surgery, when bilateral right atrial morphology was found. All patients were Asian. The study protocol was approved by local research and ethical review boards.

Information on all of the patients’ cardiac lesions was collected, including anomalous heart position, common atrium, major atrioventricular (AV) valve anomaly, pulmonary outflow obstruction, anomalous pulmonary venous connections, obstruction of pulmonary venous connections, and systemic outflow obstruction. Data on age at the first surgical intervention and types of interventions were also collected. Early mortality was defined as death occurring at any time before hospital discharge or within 30 days after the operation (even if the patient had been discharged). The day of operation was considered as day 1 for calculating the length of hospital stay. The hospital stay was considered “prolonged” after 21 days.

### Data analysis

Demographic and anatomic data, as well as procedural and outcome frequencies, were analyzed and results are presented as the number (%) of patients. Univariate analysis of the association of variables with mortality was performed using the Chi square test. A probability value of *p* < 0.05 was considered significant.

## Results

### Cardiac morphology

An abnormal heart location was observed in 13 (40 %) patients, including dextrocardia and mesocardia. Septal defects occurred in all 33 (100 %) patients, including single atrium, single ventricle, and atrioventricular septal defect. Only one patient lacked an atrial septal defect, but this patient had a large ventricular defect (>10 mm in diameter). All of the patients in this study had ventricular septal defects. Only one patient had a ventricular defect smaller than 10 mm; however, this patient also had a uniatrium. Atrioventricular valve (AVV) abnormalities occurred in 27 (87 %) patients, including a common atrioventricular valve (CAVV) and a single atrioventricular valve (SAVV) (morphologically tricuspid valve). The ventriculoarterial connection was abnormal in all 33 (100 %) patients, with the aorta originating abnormally from a univentricle, from the right ventricle, or overriding. Twenty-four (72 %) patients had a double outlet right ventricle.

Thirty-one (97 %) patients had pulmonary and (or) subpulmonary obstruction, including right ventricle outlet stenosis (57 %), pulmonary artery valve stenosis (8 %), or pulmonary atresia (9 %). Anomalous pulmonary venous drainage was observed in 21 (63 %) patients, including total anomalous pulmonary venous connection (TAPVC) and partially anomalous pulmonary venous connection (PAPVC). The incidence of these abnormalities is shown in Table 1.

**Table 1:** Anatomic cardiac findings among RAI patients

	No. of patients	Percent (%)
Cardiac position abnormal	13	39
Dextrocardia	12	36
Mesocardia	1	3
Atrioventricular septal anomaly	33	100
Atrium septum defect	32	97
Single atrium or functionally single atrium	20	61
Ventricle septum defect	33	100
Single ventricle or functionally single ventricle	19	58
Atrioventricular valve anomaly	27/31	87
SAVV (morphologically tricuspid valve)	10/31	32
SAVV (morphologically mitral valve)	1/31	3
CAVV	6/31	51
Aorta origin abnormal	33	100
From univentricle	15	45.5
From right ventricle	17	51.5
Overriding	1	3
Great arteries location abnormal	26	78
Aorta located on left of pulmonary	2	6
Right anterior	10	30
Right	4	12
Right posterior	1	2
Antterior	7	21
Pulmonary atresia	3	9
Pulmonary and (or) subpulmonary stenosis	32	97
Subpulmonary stenosis	19	57
Pulmonary stenosis	28	84
Pulmonary atresia	3	9
Anomalous common pulmonary venous trunk (ACPVT)	17	51
Anomalous pulmonary venous connection	21	63
PAPVC	6	18
TAPVC	15	45.5
BSVC	22	67

*SAVV* single atrioventricular valve, *CAVV* common atrioventricular valve, *PAPVC* partially anomalous pulmonary venous connection, *TAPVC* total anomalous pulmonary venous connection, *BSVC* bilateral superior vena cava

### Pulmonary venous drainage

In our study, 15 (45 %) patients had TAPVD, while six (18 %) had PAPVD. Pulmonary veins were observed in a variety of abnormal configurations, including connection to the right side of the atrium, to both sides of the atrium separately, to the right superior vena cava, to the left superior vena cava, to the right atrium and left superior vena cava combined, and to the left atrium and left superior vena cava combined. The incidence of these abnormalities is shown in Table 2.

**Table 2:** Pulmonary venous drainage

	No. of patients	Percent (%)
Normal pulmonary venous drainage	12	36
TAPVD	15	45
Right side of atrium	8	24
Right-SCV	2	6
Left-SCV	3	9
Part to right side of atrium and part to left-SCV	1	3
PAPVD	6	18
Both side of atrium	5	15
Part to left side of atrium and part to left SCV	1	3

*SCV* superior vena cava, *TAPVD* total anomalous pulmonary venous drainage, *PAPVD* partially anomalous pulmonary venous drainage

### Atrioventricular valve morphology and function

Twenty-seven (87 %) patients had an AVV anomaly. Sixteen (52 %) patients had CAVV and a single AV valve was observed in 11 (35 %) patients, including a morphologically tricuspid valve in 10 (91 %) patients and a morphologically mitral valve in one (9 %) patients. Echocardiography was used to estimate AV regurgitation for CAVV and SAVV cases. Table 3 shows characterization of AV regurgitation with CAVV and SAVV.

**Table 3:** Atrioventricular valve regurgitation

	CAVV n = 16	SAVV n = 11
Slight	3 (19 %)	3 (27 %)
Mild	2 (13 %)	5 (45 %)
Moderate	8 (50 %)	2 (18 %)
Severe	3 (19 %)	1 (9 %)

*CAVV* common atrioventricular valve, *SAVV* single atrioventricular valve

### Surgical interventions

Cardiovascular surgical interventions were performed in 20 (61 %) patients. Two of these (10 %) underwent a modified Blalock–Taussig shunt, including one patient who had systemic-to-pulmonary collateral coiling performed at the same time. Seven (35 %) patients underwent a bidirectional Glenn shunt, including six (30 %) who received a bilateral bidirectional Glenn shunt. Of these six patients, one received concurrent patent ductus arteriosus (PDA) ligation and pulmonary artery widening, and one underwent TAPVC repair.

Ten (50 %) patients had the total cavopulmonary connection (TCPC) procedure, including four (40 %) who underwent AVV repair at the same time. One patient had a bilateral bidirectional Glenn shunt followed by a successful TCPC procedure. No patients had biventricular repair or were prepared to have biventricular repair. The median duration of hospitalization among patients who underwent the TCPC procedure was 21 days (range: 15–90 days), with a median duration of intubation of 2 days (range: 1–8 days). Three (27 %) patients who received the TCPC procedure had prolonged hospital stay (>21 days). Reoperation during the hospital period was necessary in three (15 %) patients; two of these patients underwent TCPC and the other patient received a bilateral bidirectional Glenn shunt and PDA ligation. Only one patient died the day after operation; this patient also had a reoperation.

Of the six patients who received only TCPC, five (83 %) had an SAAV (morphologically tricuspid valve) and the other had a CAVV and died the next day after the operation. All four of the patients who underwent concurrent TCPC and AVV repair had CAVV. The patients’ degree of pre-surgical AVV regurgitation is shown in Table 4.

**Table 4:** Atrioventricular valve structure and function in TCPC patients with or without valve repair

	TCPC (n = 6)	TCPC + AVR (n = 4)
Morphology of AV valve		
SAVV (morphologically tricuspid valve)	5 (83 %)	0
CAAV	1 (17 %) early death	4 (100 %)
Degree of AV valve regurgitation		
Slight	3 (50 %)	0
Mild	3 (50 %)	0
Moderate	0	2 (50 %)
Severe	0	2 (50 %)
Early mortality	1	0

*TCPC* total cavopulmonary connection, *AVR* atrioventricular valve repair, *AV* atrioventricular, *CAVV* common atrioventricular valve, *SAVV* single atrioventricular valve

Surgical intervention was not performed in 13 (39 %) patients, with pulmonary hypertension in four (one case without pulmonary and/or subpulmonary stenosis, three cases with mild pulmonary valve stenosis), hypoplasia of the pulmonary artery in three, and moderate or severe regurgitation of the AVV in three patients.

### Comparison with previous study

Table 5 shows comparison of our study results with a previous report (Cheung et al. 2002), which discussed common abnormalities of patients with RAI. In the previous report, 89 % of patients presented during the 1st month of life. Therefore, this report described the initial abnormalities of patients with RAI. Our report described abnormalities of RAI patients who survived for at least 3 years without surgery. We found that the percentages of patients with CAVV, pulmonary vein obstruction, single or functional single ventricle, and pulmonary atresia were significantly different between the previous study and our study. There were no significant differences in the percentages of patients who were male and those who demonstrated dextrocardia/mesocardia, pulmonary/subpulmonary obstruction, or a single or functional single atrium between studies.

**Table 5:** Comparison of anatomic cardiac findings and outcome with previous reports

	This report (n = 33)	Cheung et al. (n = 116)
The time of presented RAI	More than 3 year old	76 % Within first week
Male	20 (61 %)	71/116 (61 %)
Levocardia	20 (61 %)	81/116 (70 %)
Dextrocardia/mesocardia	13 (39 %)	35/116 (30 %)
Single or functionally single atrium	20 (61 %)	69 (59 %)
Single or functionally single ventricle	19 (58 %)	96 (83 %)**
Pulmonary/subpulmonary obstruction	33 (100 %)	96 (83 %)
Pulmonary atresia	3 (9 %)	48 (41 %)**
CAVV	16/31 (51 %)	107 (92 %)**
Pulmonary vein obstruction	0	15 (13 %)*
APVD	21 (63 %)	60 (52 %)
Systemic outflow obstruction	0	4 (3 %)

*CAVV* common atrioventricular valve, *BSVC* bilateral superior vena cava, *APVD* anomalous pulmonary venous drainage, pulmonary atresia (*p* = 0.05); * *p* < 0.05; ** *p* < 0.01

## Discussion

To the best of our knowledge, this is the only report in English of a clinical series cataloging the various cardiac lesions and outcomes of patients with RAI who received surgical treatment older than 3 years of age. Despite late diagnosis, many of these patients survive surgical palliation.

## Cardiac lesions

### Anomalous pulmonary venous drainage

In our study, 15 (45 %) patients had TAPVC and six (18 %) had PAPVC. These results are consistent with previous studies (Eronen et al. 2013; Cheung et al. 2002). Sadiq et al. (1996) reported an overall survival rate of 18 % in patients requiring surgery in the 1st month of life and 78 % in patients requiring surgery after the 1st month of life. RAI is always combined with the presence of a single atrium and/or single ventricle. Therefore, patients with RAI with APVC do not need an emergent operation unless they have an obstructed anomalous pulmonary venous connection or other factors. This explains why there was no significant difference in the rate of anomalous pulmonary venous drainage between our study and the previous study.

### Pulmonary/subpulmonary obstruction

Pulmonary atresia was observed in three (9 %) patients, pulmonary stenosis in 28 (87 %) patients, and a normal pulmonary artery and pulmonary valve in two (6 %) patients. One patient with a normal pulmonary artery and pulmonary valve had right ventricular outflow stenosis. Patients with pulmonary/subpulmonary obstruction do have higher survival rates. Hashmi et al. (1998) reported that the absence of pulmonary outflow obstruction was an independent risk factor for earlier death. Previous studies have shown that 8–15 % of patients with RAI need pulmonary artery banding (PAB), and mortality of these patients with PAB is 75–95 % (Eronen et al. 2013; Cheung et al. 2002; Ota et al. 2012). This could explain why few patients with pulmonary atresia were observed in our study. In our study, there was one patient without pulmonary and subpulmonary obstruction. This patient and three other patients with mild pulmonary stenosis lost the opportunity for surgical interventions.

### Obstructed anomalous pulmonary venous connection

Previous studies have indicated that 25–45 % of patients with RAI also have an obstructed anomalous pulmonary venous connection (Cheung et al. 2002; Sadiq et al. 1996; Yun et al. 2006). In our study, however, no patients had an obstructed anomalous pulmonary venous connection. An obstructed anomalous pulmonary venous connections is a poor prognostic indicator in RAI (Sadiq et al. 1996; Phoon and Neill 1994). The surgical mortality rate in patients with pulmonary vein repair is 95–97 % and it is thought to be an independent risk factor for early death (Jenkins et al. 1993). Patients with an obstructed anomalous pulmonary venous connection are unlikely to survive beyond 3 years of age. Therefore, this could explain the significant difference in the number of patients with an obstructed anomalous pulmonary venous connection in our study compared with previous studies. Our study further confirms the risk of RAI with obstructed anomalous pulmonary venous connection.

### Atrioventricular valve morphology and function

AVV morphology and function are important factors when considering surgery. In our study, although the total proportion of patients with an abnormal AVV was similar to that in previous studies (Cheung et al. 2002), the proportion of CAVV was significantly lower. Previous studies (Cheung et al. 2002) have shown that the incidence of CAVV in RAI is 75–100 %. In our study, we found that all patients undergoing TCPC and AVV repair had a CAVV. All of the patients undergoing TCPC without AVV repair had an SAVV, except for one patient who died soon after the operation. This finding suggests that CAVV was likely associated with moderate or severe regurgitation, leading to earlier AVV repair.

AVV regurgitation in RAI is primarily caused by structural abnormalities, including abnormal clefts and commissures, dysplastic leaflets, and elongated or shortened chordae (Uemura et al. 1998; Kawahira et al. 1997). Ota et al. (2012) reported that 13 (21.7 %) patients with RAI had severe systemic AVV regurgitation on their first echocardiogram. Perioperative management with intubation and nitrogen inhalation reduced the AVV regurgitation in five (8 %) of these patients, and the remaining eight (13 %) patients underwent valve repair at their initial palliative operation. Persistent AVV regurgitation, which is regurgitation that persists or recurs after attempted valve repair, is a potential risk factor for mortality (Hashmi et al. 1998). The peculiar morphological characteristics of the AVV may be the cause of frequent valvar regurgitation in patients with RAI (Francalanci et al. 1996; Lin et al. 2002). AVV regurgitation leads to an enlarged ventricle and atrioventricular annulus, further increasing atrioventricular regurgitation with age. This could explain why a high number of patients required AVV repair in our study. In our study, we observed that CAVV regurgitation was more problematic than SAVV.

### Bilateral superior vena cava

In our study, 22 (67 %) patients had bilateral superior vena cava. This rate is much higher than that observed in a previous study (26 %) (Ota et al. 2012). The reason for this difference in findings is unclear. No previous studies have demonstrated that a single superior vena cava is a risk factor for early death. We suspect that pulmonary venous drainage obstruction is more likely in patients with only one superior vena cava than in patients with bilateral superior vena cavas. Therefore, the presence of bilateral superior vena cava may reduce obstruction of pulmonary venous drainage.

### Systemic outflow stenosis

Previous studies have shown an incidence of 5–12 % of systemic outflow stenosis in patients with RAI (Eronen et al. 2013; Ota et al. 2012; Kawahira et al. 1997). Systemic outflow stenosis may increases the difficulty of the operation. And the infant mortality rate within this group is nearly 100 % (Ota et al. 2012). This could explain why patients with systemic outflow obstruction were not found in our study.

### Study limitations

Our institution, Fuwai Hospital,is a specialty institution with Pediatric Cardiac Surgical Center for congenital cardiac patients. However, we do not have newborn service in this hospital. Therefore, no prenatal or neonatal patients were enrolled into this study. This made it difficult to determine some risk factors for a poor outcome in patients with RAI. Several genes are responsible for left–right laterality and heterotaxy syndrome, including *ZIC3*, *NODAL*, and *LEFTY2* (Belmont et al. 2004; Zhu et al. 2006). Despite high infant mortality rates, some patients with RAI can live to at least 3 years of age without surgery. We suspect that genetic differences may lead to this anatomy, but we did not perform any genetic analysis in our study.

## Conclusion

Our study reports that some RAI patients can survive beyond 3 years of age without surgical intervention despite having complex cardiac abnormalities. In older RAI patients, survival after single stage total cavopulmonary connection is good. CAVV regurgitation is more serious than SAVV regurgitation and it frequently needs to be repaired. Further studies, especially genetic studies, are warranted to improve the long-time results for these older patients.
